# Integrated RNAi screening identifies the NEDDylation pathway as a synergistic partner of azacytidine in acute myeloid leukemia

**DOI:** 10.1038/s41598-021-02695-0

**Published:** 2021-12-02

**Authors:** Justine Klosner, Konstantin Agelopoulos, Christian Rohde, Stefanie Göllner, Christoph Schliemann, Wolfgang E. Berdel, Carsten Müller-Tidow

**Affiliations:** 1 Department of Medicine A, Hematology, Oncology and Pneumology, University Hospital Münster, Münster, Germany; 2 Department of Medicine V, Hematology, Oncology and Rheumatology, University Hospital Heidelberg, Heidelberg, Germany; 3 Department of Dermatology and Center for Chronic Pruritus, University Hospital Münster, Münster, Germany

**Keywords:** Acute myeloid leukaemia, Molecular medicine

## Abstract

Treatment of acute myeloid leukemia (AML) remains challenging and novel targets and synergistic therapies still need to be discovered. We performed a high-throughput RNAi screen in three different AML cell lines and primary human leukemic blasts to identify genes that synergize with common antileukemic therapies. We used a pooled shRNA library that covered 5043 different genes and combined transfection with exposure to either azacytidine or cytarabine analog to the concept of synthetic lethality. Suppression of the chemokine CXCL12 ranked highly among the candidates of the cytarabine group. Azacytidine in combination with suppression of genes within the neddylation pathway led to synergistic results. NEDD8 and RBX1 inhibition by the small molecule inhibitor pevonedistat inhibited leukemia cell growth. These findings establish an in vitro synergism between NEDD8 inhibition and azacytidine in AML. Taken together, neddylation constitutes a suitable target pathway for azacytidine combination strategies.

## Introduction

Acute myeloid leukemia (AML) is the most common type of acute leukemia in adults. AML is defined by a bone marrow infiltration of at least 20% of undifferentiated or abnormally differentiated clonal, myeloid progenitor cells. Although it occurs at all ages, the majority of patients are older than 60 years at diagnosis^1^. The standard treatment for AML patients medically fit for intensive chemotherapy consists of induction chemotherapy followed by either consolidation chemotherapy or allogeneic stem-cell transplantation^2^. Unfortunately, elderly patients are often not eligible for intensive chemotherapy due to age, overall performance status, and comorbidities. Furthermore, the high incidence of adverse genetic abnormalities in patients older than 60 years of age results in complete response rates inferior to younger patients (40–60% versus 60–85%)^3,4^. For these patients, low-dose cytarabine or hypomethylating agents (5-azacytidine and decitabine) are the standard of care. However, these low-intensity regimens yield low response rates with a median overall survival of only 10.4 months and have to be considered as palliative treatment^5,6^.

Recently, the therapeutic landscape in AML has changed. New drugs were approved including targeted therapies and venetoclax combinations^7–9^. The addition of the BCL-2 inhibitor venetoclax to azacytidine leads to higher complete remission rates and overall survival in patients unfit for intensive chemotherapy, which demonstrates the synergistic potential of adding targeted therapies to standard compounds. However, relapses occur frequently, overall survival rates are still dismal and new therapeutic targets are needed.

Aiming to identify new therapeutic targets whose inhibition sensitizes AML cells towards the two standard therapeutic agents cytarabine and azacytidine we performed an in vitro synthetic lethality RNAi screen.

RNA interference (RNAi) screens with pooled shRNA libraries have been a reliable strategy for identifying new therapeutic targets^10,11^. Aside from the identification of potential therapeutic targets, RNAi screens can also be performed as chemosensitization screens. By exploiting the concept of synthetic lethality, new drug-combinations with synergistic effects can be identified when combining an RNAi screen with standard chemotherapy^12–15^. To this end, we conducted a classic dropout viability screen in combination with a minimal dose of either azacytidine or cytarabine to uncover targets whose depletion decreases viability in presence of the drugs. Since AML is a genetically very heterogeneous disease^16^, three different AML cell lines as well as primary human leukemic cells were used in order to yield robust results.

## Results

### RNAi screen design and analysis

**Figure 1:**
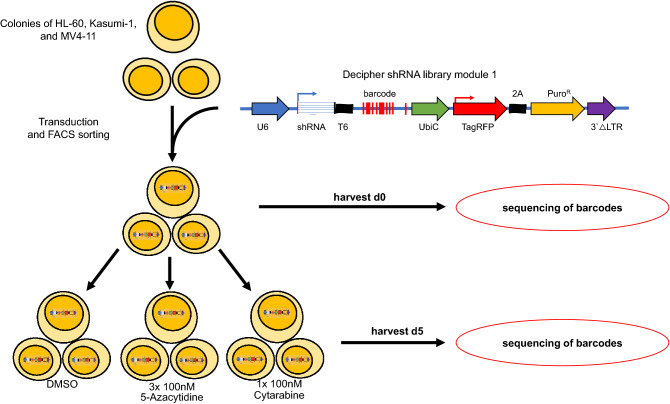
Pooled RNAi screens in leukemia cell lines and primary blasts. The in vitro shRNA screen was performed in three different cell lines and in primary leukemia blasts, resulting in ten independent experiments. Successfully transduced cells were selected via FACS sorting for TagRFP, expanded and subsequently exposed to either cytarabine, azacytidine or DMSO for 72 h. Cells were harvested at day 0 and day 5, genomic DNA was extracted and shRNA abundance was analyzed by next generation sequencing.

The RNAi screen was performed in HL-60-, Kasumi-1- and MV4-11-cells. Transduced cells were exposed to either azacytidine or cytarabine in sublethal concentrations to identify candidates for synthetic lethality (Supplemental Fig. 1). shRNA representation was analyzed at day 0 and day 5 using parallel sequencing of PCR-amplified shRNA barcodes from extracted genomic DNA (Fig. 1). Results were normalized to the median read count of day 0. For further analysis, data of all three cell lines were combined to identify candidates with universal synergistic results. Overall, 10 independent experiments were performed leading to a total of 412,500 data points. Due to the complex data matrix we decided to identify meaningful candidate genes based on approaches that were commonly used to analyze microarray data. Read counts for each shRNA and for each experiment were combined and uploaded into BRB ArrayTools, an R-based software with Excel-front end. Candidates were determined by class comparison in BRB ArrayTools, hits were further filtered by p-value (p < 0.05) and depletion rate (fold change < 0.6). Genes for which at least two independent shRNAs were depleted were counted as potential targets.

### RNAi screen results

The screen targeted more than 5000 genes, among them were several positive controls and 21 shRNAs targeting Luciferase as a negative control. Our approach allowed us to filter for genes that increased the cells’ sensitivity towards cytarabine and azacytidine.

**Figure 2:**
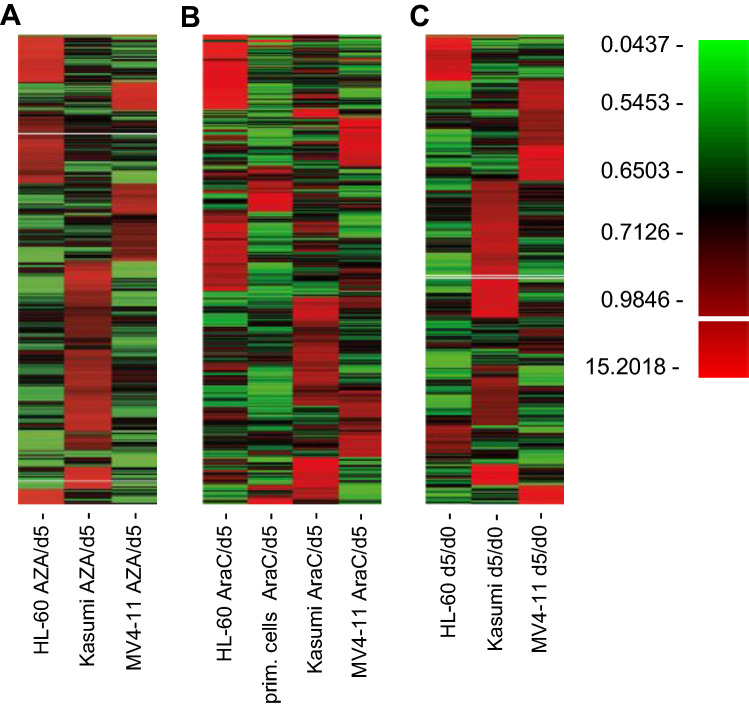
Screening heterogenous cell lines leads to only few potential targets. (**A**) Heatmap showing the alterations of representation of 27,500 shRNAs in HL-60, Kasumi-1 and MV4-11 exposed to sublethal concentrations of azacytidine (AZA) at day 5. (**B**) Heatmap showing the alterations of representation of 27,500 shRNAs in HL-60, Kasumi-1, MV4-11 and primary human blasts exposed to sublethal concentrations of cytarabine (AraC) at day 5. (**C**) Heatmap depicting changes in representation of 27,500 shRNAs over the course of 5 days in HL-60, Kasumi-1 and MV4-11.

Aside from the overall analysis we also examined the results of each cell line independently and compared them with each other (Fig. 2A–C). As expected, results for each cell line differed drastically from each other, emphasizing the heterogenous results of each cell line.

**Figure 3:**
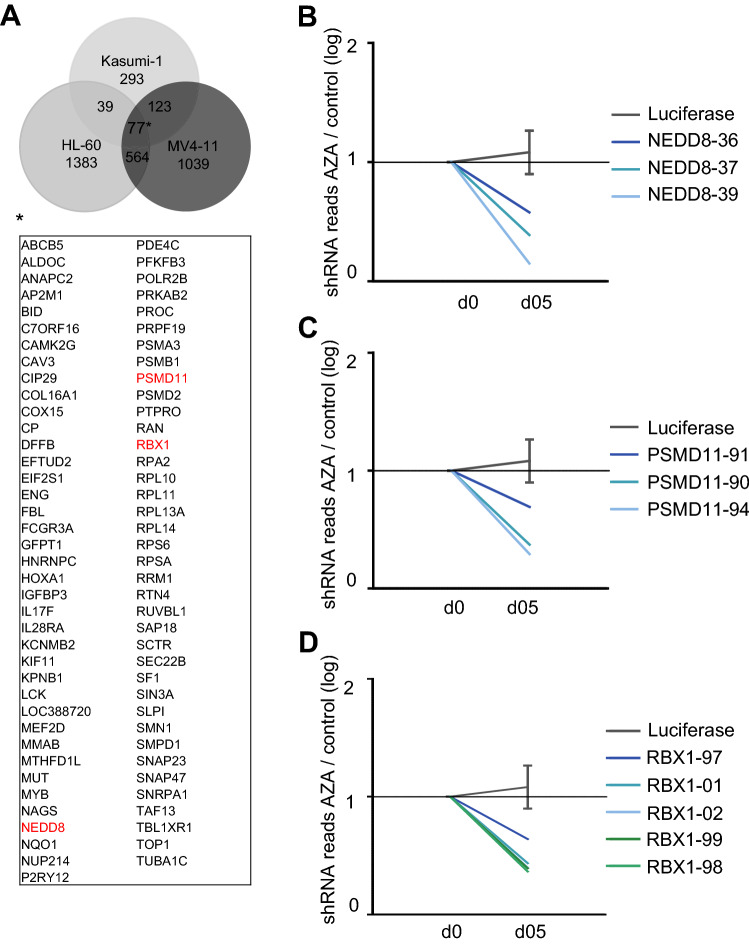
Comparison of results in the Azacytidine group. (**A**) Venn diagram shows the number of potential candidates in each tested cell line in the AZA/d05 analysis and visualizes mutually depleted genes (fold change < 0.6) with at least two independent shRNAs targeting the same gene. Among them are NEDD8, RBX1 and PSMD11, all part of the proteasome complex. (**B**–**D**) Plots showing median depletion of different shRNAs targeting NEDD8 (**B**), PSMD-11 (**C**) and RBX-1 (**D**) in HL-60, Kasumi-1 and MV4-11.

Analysis of the data of the azacytidine group yielded 77 mutually depleted genes as potential candidates. Interestingly we found three genes involved in the proteasome cell compartment, namely NEDD8, RBX1 and PSMD11. Most significant depletion was achieved by the shRNAs targeting NEDD8 with a log-fold change of − 0.82 (Fig. 3B); shRNAs targeting NEDD8 were depleted in all cell lines.

**Figure 4:**
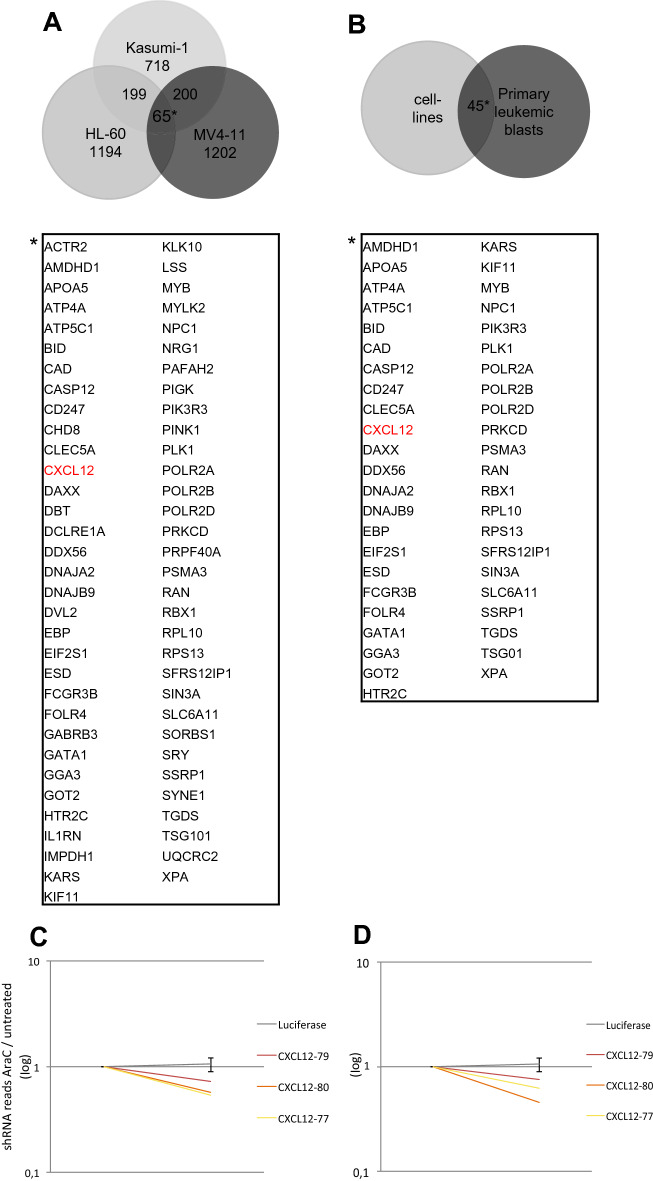
Comparison of results in the cytarabine group and primary cells. (**A**) Venn diagram shows the number of potential therapeutic targets in each tested cell line in the AraC/d05 analysis and visualizes mutually depleted genes (fold change < 0.6) with at least two independent shRNAs targeting the same gene. Among the 65 hits is CXCL12. (**B**) Venn diagram shows mutually depleted genes of cell lines (HL-60, Kasumi-1, MV4-11) and primary leukemic blasts when comparing AarC/d05 ratio. Again, only targets with two independent shRNAs were taken into account. (**C**,**D**) Plots showing median depletion of different shRNAs targeting CXCL12 in cell lines (**C**) and in primary leukemic blasts (**D**).

For cells exposed to cytarabine, we identified 65 mutual candidates that were significantly depleted with a negative fold change of < 0.6. CXCL12 was identified as an enhancer of cytarabine activity (Fig. 4A,C). This is in line with previous reports and suggests that valid results could be obtained by our screening approach^17,18^. Matching results of cell lines and primary leukemic blasts produced 45 mutual candidates, which again included our positive control CXCL12 (Fig. 4B,D).

Taken together our large-scale screening approach revealed an overlap of shared potential targets, including positive controls, within all three cell lines.

### Suppression of the neddylation pathway sensitizes AML cells towards azacytidine in sublethal concentrations

**Figure 5:**
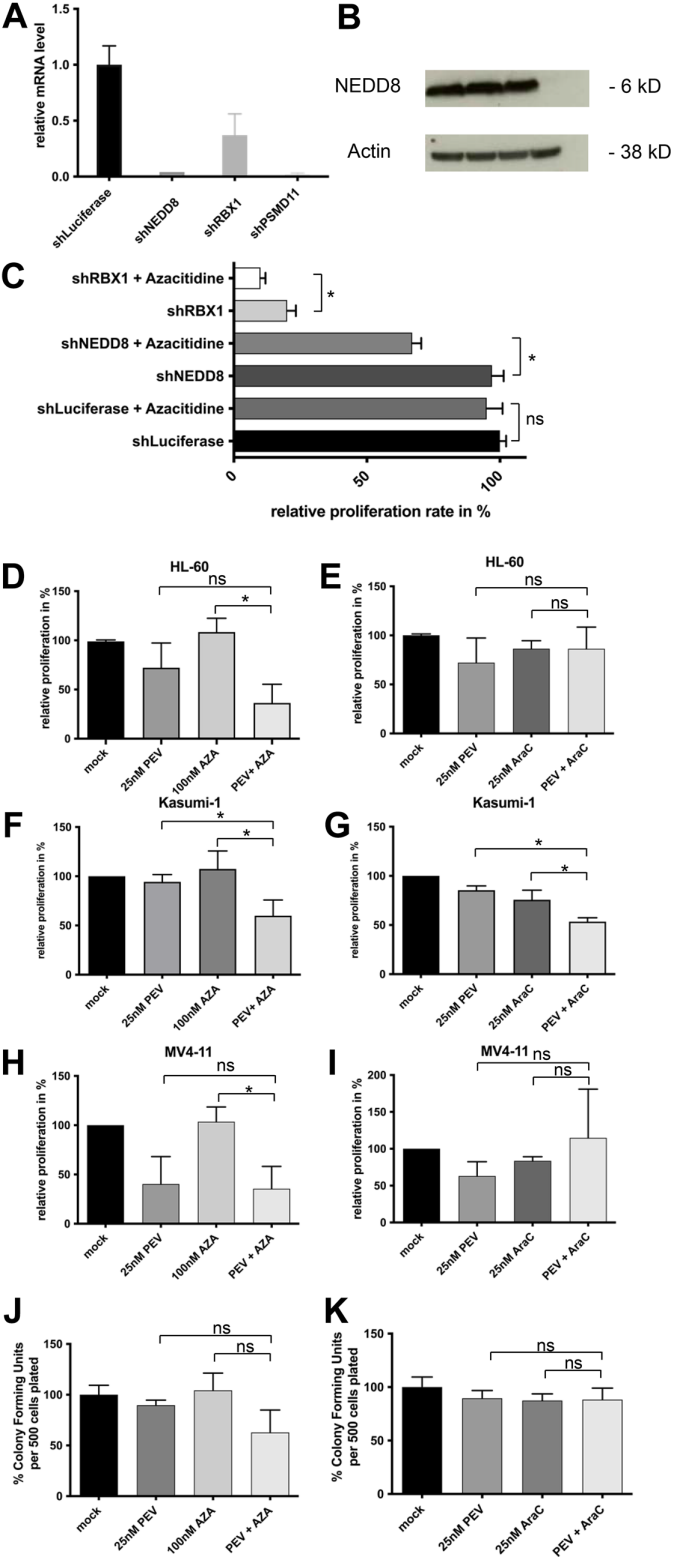
Validation of NEDD8 as a synergistic target in AML. (**A**) mRNA levels of NEDD8, RBX1, and PSMD11 were assessed in HL-60 cells transfected with corresponding shRNA and control shRNA. (**B**) Protein levels were measured in HL-60 with Western blotting of whole-cell lysates. (**C**) Proliferation rates of HL-60 cells transfected with shRNAs targeting either NEDD8 or RBX1 and Luciferase as control. Transfected cells were exposed to 500 nM azacytidine (AZA) or DMSO for 24 h. Proliferation rates were calculated by cell counting after 72 h and normalized to the proliferation rate of DMSO treated cells. (**D**–**I**) Proliferation rate of HL-60 and Kasumi-1 exposed to NEDD8 inhibitor pevonedistat (PEV) in combination with either azacytidine (AZA) or cytarabine (AraC). Proliferation rate was measured by cell counting and calculated as explained above. (**J**–**K**) Methylcellulose colony-forming assays were performed in HL-60 cells after 72 h daily treatment in vitro with either azacytidine (AZA) or cytarabine (AraC) in combination with pevonedistat (PEV). Controls were treated with DMSO.

Three proteasome pathway genes were identified in our screen with two of those (NEDD8 and RBX1) both being members of the neddylation pathway. In addition, NEDD8 provides a rationale target since a small molecule inhibitor (MLN4924/pevonedistat) is already used in clinical trials^19,20^. To validate the potential candidates, we first analyzed the knockdown efficiency of the strongest depleted shRNA from the screen. Aside from NEDD8 we also sought to check for the knockdown efficiency of other shRNAs for quality control of the screening results (Fig. 5A). First, knockdown efficiency was confirmed by quantitative real-time PCR and western blot (Fig. 5A,B, Supplemental Fig. 2). Next, we sought to confirm the synergistic effect of combining NEDD8 knockdown with exposure to azacytidine. Suppression of NEDD8 and RBX1 in HL-60 cells notably sensitized cells towards azacytidine, whereas azacytidine alone failed to inhibit the proliferation rate of HL-60 cells transfected with the negative control shRNA targeting Luciferase (Fig. 5C).

Next, we examined whether we could confirm these findings with pevonedistat (PEV), a small-molecule inhibitor of the NEDD8 activating enzyme (NAE). Proliferation of AML cell lines was disrupted by the combination of pevonedistat and azacytidine (AZA). (Fig. 5D–I). To exclude that diminished proliferation rates are only due to unspecific cytotoxic effects of drug addition, we also combined pevonedistat with cytarabine (AraC). The efficacy of the combination AZA/PEV in comparison to AraC/PEV was most pronounced in HL-60. In MV4-11 the combination AZA/PEV was also more efficient than in AraC/PEV, whereas in Kasumi-1 the effect was similar in both groups. Of note, the efficacy of the combination was also most pronounced in HL60 cells in the initial screen.

The combination of azacytidine and pevonedistat was also efficient to inhibit subsequent colony formation in methylcellulose assays whereas the combination of pevonedistat and cytarabine failed to mimic this effect (Fig. 5J–K).

## Discussion

Despite recent advances in the treatment of acute myeloid leukemia, efficacy of therapy remains an important problem. To identify new potential therapeutic combinations, we conducted a large-scale RNAi screen. Comparison of the cell lines revealed only a small amount of overlapping potential targets (Figs. 2, 3, 4), thus screening several cell lines at once appears to be a useful strategy to discover promising new therapeutic approaches. Further, our screening approach allowed us to analyze a variety of aspects, e.g., antiproliferative targets and targets with synergistic effects in combination with either cytarabine or azacytidine.

We included primary human AML cells in our cytarabine screen. As already shown, RNAi screens in primary cells are feasible^21^. Our simultaneous screen of leukemic cell lines and primary human leukemic cells was followed by a comparison of cell lines and primary cells, which ranked CXCL12 among the top targets. The chemokine CXCL12 and its corresponding receptor CXCR4 on hematopoietic stem cells (HSCs) are key mediators of homing of HSCs to the bone marrow and are involved in the homeostasis of the HSCs pool—a mechanism exploited by leukemic cells. Elevated CXCR4 expression correlates with a poor prognosis in AML and suppression of CXCR4 can promote sensitivity to cytarabine^18,22–24^. In recent years small molecule inhibitors, peptide inhibitors, and anti-CXCR4 monoclonal antibodies have been developed and tested to target the CXCL12-CXCR4-axis yielding first promising results^25,26^.

**Figure 6:**
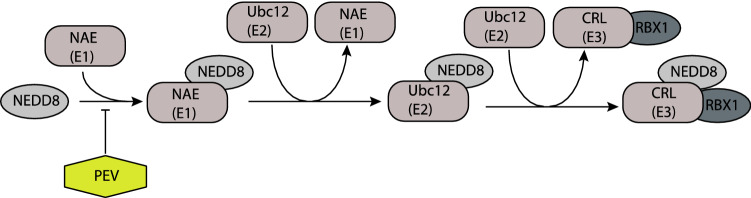
Neddylation pathway and its inhibitor pevonedistat. The activation of the largest ubiquitin ligases subgroup cullin-ring ligases (CRLs) starts with activation of NEDD8 by the NEDD8 E1 activation enzyme (NAE) in an ATP-dependent reaction. Afterwards, it transfers to the conjugation enzyme E2. The NEDD8 charged E2 then conjugates NEDD8 to a cullin-containing RING finger ligase (CRL). The neddylation pathway can be disrupted by the small molecule inhibitor pevonedistat, which inhibits NAE.

Among our top candidates for azacytidine were NEDD8 and RBX1 which both belong to the ubiquitin–proteasome-system. NEDD8, a ubiquitin-like protein, activates cullin-Ring ligases (CRLs), which are a large subgroup of E3 ubiquitin ligases (Fig. 6)^27–29^. RBX1 is also involved in the ubiquitin–proteasome system and is the RING component of CRLs, a large subclass of E3 ubiquitin ligases. CRL activity is promoted by cullins, which function as molecular scaffolds and are the best-characterized NEDD8 substrates^30,31^.

In further in vitro experiments we observed that inhibiting the Neddylation pathway by combining pevonedistat and sublethal doses of azacytidine disrupted the proliferation. These findings suggest that inhibition of the neddylation pathway sensitizes AML cells towards azacytidine. Also, pevonedistat in combination with azacytidine was able to reduce the clonogenic ability of AML cells. In most experiments this effect could not be reproduced in combination with cytarabine, suggesting a specific mechanism.

Consistent with our findings, aberrant ubiquitylation and neddylation have been linked to the development of cancer and specifically to AML^32^. The proteasome inhibitor bortezomib is a successful example of the therapeutic effects of inhibition of the proteasome complex in malignant diseases^33–35^. We recently showed that proteasome inhibition by bortezomib overcame a multi drug resistance phenotype in AML^36^. Meanwhile, there are ongoing clinical investigations for pevonedistat (MNL4929) combination therapies^20,37,38^. Of note, it was previously also shown that azacytidine can help overcome pevonedistat resistance by antagonizing pevonedistat-induced Ribonucleotide Reductase subunit 2 (RRM2) increase^39^.

Both azacytidine and cytarabine are cytidine analogs and are incorporated into DNA, functioning as antimetabolites. Aside from cytotoxic effects, azacytidine also induces DNA demethylation by inhibiting DNA Methyltransferase 1 (DNMT1) after being incorporated into the DNA and nanomolar doses can reduce self-renewing and leukemia-initiating capacities without leading to direct cytotoxic effects^40–42^. Nonetheless, the exact mechanism of action of azacytidine is not completely understood.

One limitation of our study is the lack of in vivo experiments as well as further functional studies. Accordingly, further data need to be acquired. However, preclinical data to support a specific synergism of pevonedistat and azacytidine is scarce.

Taken together, our data suggests that suppression of the neddylation pathway sensitizes AML cells towards sublethal doses of azacytidine. The exact mechanism remains to be elucidated and clinical studies shall further establish the clinical benefit of this combination.

## Methods

### Cell culture

Human leukemic cell lines were purchased from Deutsche Sammlung von Mikroorganismen und Zellkulturen (DSMZ, Braunschweig, Germany). HL-60 and Kasumi-1 were cultured in RPMI 1640 (Sigma-Aldrich, Darmstadt, Germany) supplemented with 10% fetal bovine serum (Biochrom, Berlin, Germany), 1% penicillin–streptomycin (PAA laboratories, Pasching, Austria) and 1% l-Glutamine (Sigma-Aldrich). MV4-11 and HEK293T were cultured in IMDM (Invitrogen, Carlsbad, CA, USA), also supplemented with 10% fetal bovine serum, 1% penicillin–streptomycin and 1% l-Glutamine. All cell lines were regularly tested for mycoplasma contamination.

### Primary AML cells

Primary cells were obtained from peripheral blood at the time of relapse with informed consent of the donor according to protocols approved by the institutional review boards (Ethik-Kommision der Ärztekammer Westfalen-Lippe und der Medizinischen Fakultät der Westfälischen Wilhelms Universität Münster, AZ 2007-390-f-S) and in accordance with the Declaration of Helsinki. Cells were grown in RPMI, supplemented with 20% fetal bovine serum and 5% giant cell tumor conditioned medium (Irvine Scientific, Santa Ana, CA, USA).

### Chemicals

Cytarabine, azacytidine and pevonedistat (MLN4924) were purchased from Sigma-Aldrich. All drugs were stored at − 20 °C.

### Barcoded negative-selection RNAi screen

The Decipher lentiviral shRNA library module 1 (Cellecta, Mountain View, CA, USA), covering 5043 genes, contained 27,500 shRNAs in a lentiviral backbone with appropriate positive and negative controls. The library was designed to target each gene with 5 different shRNAs.

For production of lentiviral particles were produced by HEK293T cells. HEK293T cells were transfected with 6 µg of lentiviral plasmid and ViraPower™ helper plasmids (Life Technologies, Darmstadt, Germany) using Lipofectamine 2000 (Invitrogen, Carlsbad, CA, USA). Supernatants containing viral particles were harvested after 48 h and afterwards filtered through sterile syringe filters (Thermo Fisher Scientific, Waltham, MA, USA) and stored at − 80 °C.

For lentiviral transduction dishes were coated with RetroNectin^®^ (Fisher Scientific, Waltham, MA, USA). To guarantee sufficient library representation total number of 30 million cells were used for transduction and at least a quantity of 20 million was maintained during the experiment. 72 h after transduction, infected cells were selected by cell sorting using a BD FACSAria™ III (BD Biosciences, San Jose, CA, USA) and subsequently expanded for approximately further 72 h until required cell number was reached. At this time point, 20 million cells were harvested (day 0) and frozen at − 20 °C. Remaining cells were split into three treatment groups and exposed to either 100 nM azacytidine, 100 nM cytarabine or to DMSO for 72 h. After treatment cells were kept in culture for further 48 h and harvested on day 5. Genomic DNA from samples harvested at day 0 and day 5 was extracted using the DNeasy Blood and Tissue Kit (Qiagen, Hilden, Germany) according to the manufacturer’s protocol. DNA was further concentrated by ethanol precipitation. Next, two rounds of PCR for barcode amplification followed as described elsewhere^43^. Before sequencing analysis, samples were marked with a specific barcode and multiplexed with Encore TM 384 Multiplex System (NuGen Technologies, Leek, NL). A total of 12 pmol gDNA was used. Decipher barcode abundance was analyzed by next-generation sequencing with a HiScanTMSQ System (Illumina, San Diego, USA). Next-generation sequencing was carried out with a mean coverage of 250 reads per shRNA and 8 million reads per sample.

### Quantitative real-time PCR (qPCR)

To analyze expression of mRNA HEK293T cells were transduced with highest scoring shRNA for PSMD11, NEDD8 and RBX1. RNA was isolated using RNeasy Mini Kit (Qiagen, Hilden, Germany). Quantitative PCR was performed according to manufacturer’s instructions with SYBR green. All signals were normalized to levels of *Gapdh* and results were calculated by the ΔCt method.

### Western blot analysis

Positively transduced cells were lysed using the radioimmunoprecipitation assay (RIPA) buffer. SDS-PAGE and western blotting were performed as already described^44^. Antibodies against NEDD8 and RBX1 were obtained from Cell Signaling Technology (Danvers, MA, USA), an antibody against PSMD11 was obtained from Novus Biologicals (Littleton, CO, USA).

### Proliferation assays

Cell proliferation assays were performed by cell counting or by MTS assays. All assays were done in triplicate. MTS assays (Promega Corp., Fitvhburg, WI USA) were used for the determination of IC_50_. HL-60, Kasumi-1 and MV4-11 were treated with azacytidine or cytarabine from 5 to 1000 nm. Cell proliferation was measured after 48 h with a Benchmark microplate reader (Bio-Rad, Hercules, CA, USA).

In vitro shRNA Proliferation assays were performed with HL-60, Kasumi-1, and MV4-11. Transduced cells were selected as described above and exposed to 100 nM azacytidine for 72 h. For inhibitor testing, cells were exposed to 25 nM pevonedistat in combination with either 100 nM, 500 nM azacytidine or 25 nM cytarabine for 72 h. Subsequently, cells were transferred to fresh medium. Viable cells were counted at day 5 using the TC20TM Automated Cell Counter (Bio-Rad, Hercules, CA USA), dead cells were excluded by Trypan Blue staining. Relative proliferation rates were calculated by normalizing to mock-treated cells.

### Methylcellulose colony-forming assay

HL-60 cells were exposed to pevonedistat in combination with azacytidine or cytarabine in a 3-day drug treatment scheme; medium was exchanged every 24 h. An equal number of cells (500 per plate) were plated in triplicates on to methylcellulose M0512 (Sigma-Aldrich) supplemented with fetal bovine serum and l-Glutamine. Colonies containing more than 40 cells were scored after 7 days.

### Statistical analysis

Raw data of sequencing analysis was first exported using the software Illumina CASAVA Software Version 1.8.2. Next sequences were analyzed by the “Barcode Deconvoluter” software provided by Cellecta. Further analysis was performed in BRB ArrayTools, an R-based software with Excel-front end.

Statistical differences were analyzed by Student’s t-test using GraphPad Prism software. A p-value < 0.05 was defined as a significant difference.

## Supplementary Information

Supplementary Figure 1.

Supplementary Figure 2.

Supplementary Legends.
