# Supplementary figures and images for: Integrated RNAi screening identifies the NEDDylation pathway as a synergistic partner of azacytidine in acute myeloid leukemia
Source: Sci Rep. 2021 Dec 2;11:23280. doi: 10.1038/s41598-021-02695-0 (PMC8639713; doi:10.1038/s41598-021-02695-0)

## Slide 1
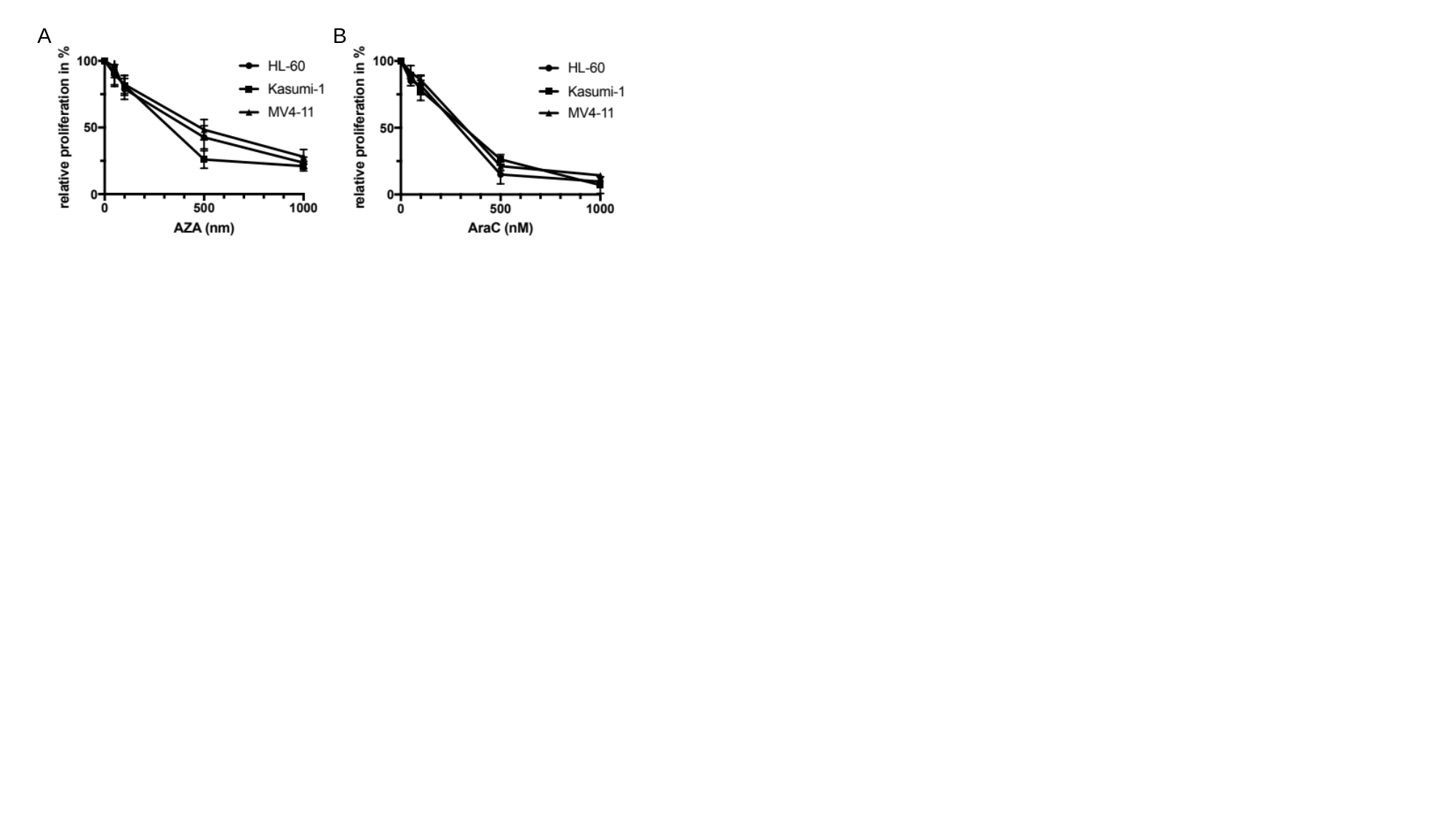

A
B

Supplement: Supplementary file 1 — Supplementary Figure 1. [file 41598_2021_2695_MOESM1_ESM.pptx]

## Slide 1
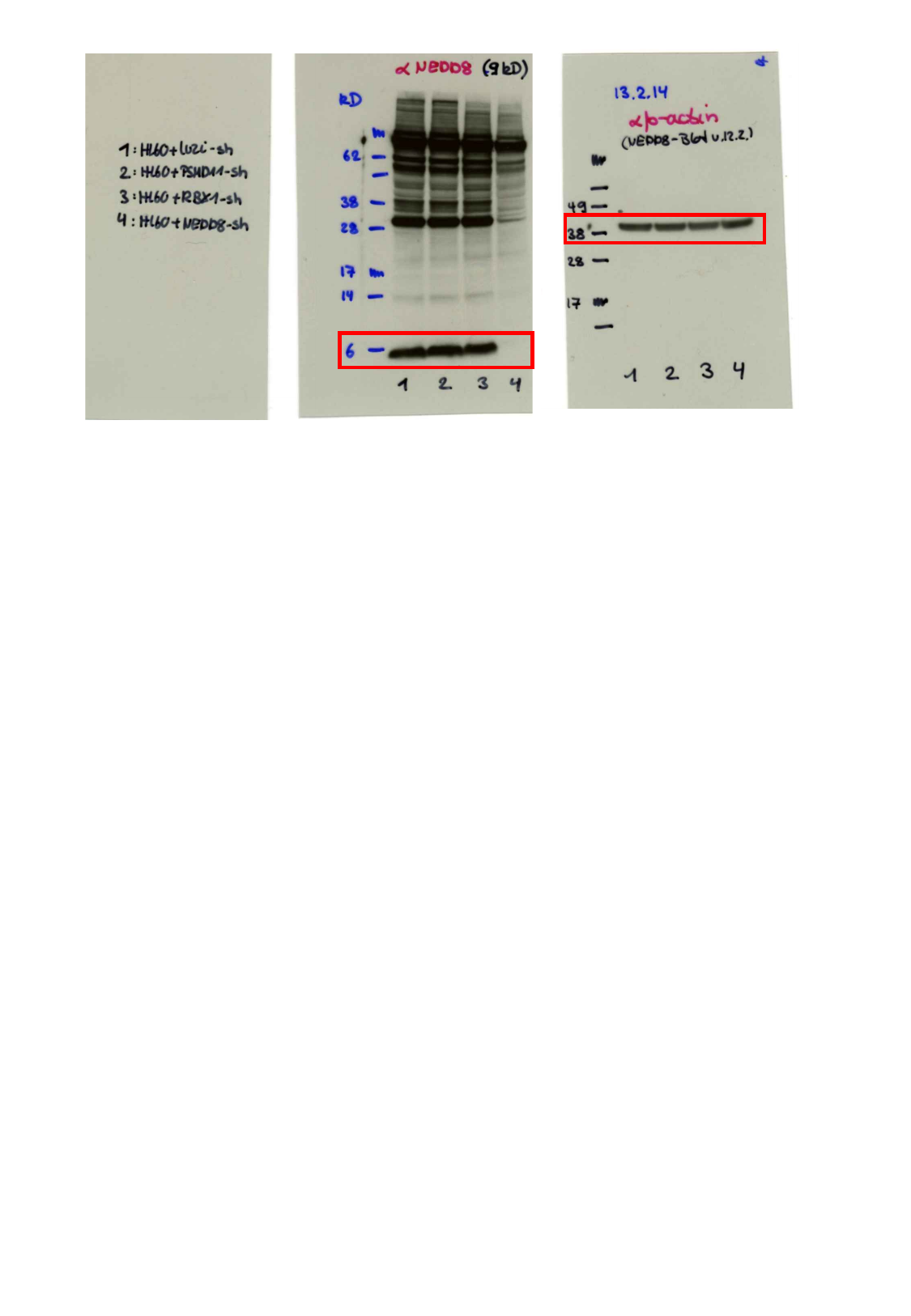

Supplement: Supplementary file 2 — Supplementary Figure 2. [file 41598_2021_2695_MOESM2_ESM.pptx]
