# Supplementary material for: Integrated RNAi screening identifies the NEDDylation pathway as a synergistic partner of azacytidine in acute myeloid leukemia
Source: Sci Rep. 2021 Dec 2;11:23280. doi: 10.1038/s41598-021-02695-0 (PMC8639713; doi:10.1038/s41598-021-02695-0)
Supplement: Supplementary file 3 — Supplementary Legends. [file 41598_2021_2695_MOESM3_ESM.docx]

Supplemental Figure 1. Establishing drug concetrations for IC_20_

(A)-(B) Proliferation assays to establish IC_20_ were performed in HL-60 cells. Cells were exposed to different doses of either azacytidine or cytarabine and harvested after 72h. Proliferation rate was analyzed by cell counting.

Supplemental Figure 2. Western blot analysis

Protein levels were measured in HL-60 with Western blotting of whole-cell lysates. This figure shows the uncropped gel blot of Figure 5B.
